# Triplet therapy overcomes 3rd-EGFR TKI-resistant EGFR-L858R/T790M/C797S in trans and in cis/L718Q mutation

**DOI:** 10.1016/j.gendis.2024.101408

**Published:** 2024-09-07

**Authors:** Kaibo Ding, Zhongsheng Peng, Yanjun Xu

**Affiliations:** Department of Medical Thoracic Oncology, Zhejiang Cancer Hospital, Hangzhou Institute of Medicine (HIM), Chinese Academy of Sciences, Hangzhou, Zhejiang 310022, China; Postgraduate Training Base Alliance of Wenzhou Medical University (Zhejiang Cancer Hospital), Hangzhou, Zhejiang 310022, China; Department of Medical Thoracic Oncology, Zhejiang Cancer Hospital, Hangzhou Institute of Medicine (HIM), Chinese Academy of Sciences, Hangzhou, Zhejiang 310022, China

Epidermal growth factor receptor (EGFR)-positive non-small cell lung cancer is a dynamic entity, with tumor progression and resistance to tyrosine kinase inhibitors (TKIs) stemming from the accumulation of mutations over time and across different disease sites, leading to temporal and spatial heterogeneity.^1^ Based on the presence of EGFR T790M and the allelic background of C797S-T790M, four subgroups of patients after three-generation EGFR-TKI resistance can be distinguished: EGFR T790M-C797S in cis subtype; EGFR T790M-C797S in trans subtype; EGFR T790M-C797S in trans and cis subtype; and wild-type EGFR codon T790M but carrying the C797S mutation, hereinafter referred to as C797S-only.^2^ While treatment modalities have emerged for the different mutant subtypes, a recent study has found that the prognosis for the EGFR T790M-C797S in trans and cis subtype and the C797S-only subtype remains poor.^2^ This may be due to dynamic changes in EGFR clones under therapeutic stress. Here, we report data for the evolution of the EGFR mutation in a patient with non-small cell lung cancer during disease progression under the pressure of EGFR-TKI therapy. In the patient's latest genetic test, we identified the coexistence of five different EGFR mutations, including EGFR L858R, EGFR T790M-C797S both in cis and in trans and EGFR L718Q. Then she was treated with a triplet therapy of EGFR-TKIs, and stabled for three months.

In December 2017, a 43-year-old female patient with no history of smoking underwent a lumpectomy for radical treatment of adenocarcinoma in the upper left lung at an external institution and received four cycles of postoperative adjuvant treatment of pemetrexed and cisplatin (postoperative pathologic staging: pT1bN1M0, ⅡB). After two years, the patient was referred to our hospital with persistently elevated carcinoembryonic antigen (CEA). The patient's positron emission tomography–computed tomography (PET-CT) scan revealed multiple fluoro-2-deoxy-d-glucose (FDG)-positive mediastinal lesions in the lymph nodes of the chest. Postoperatively, the patient was diagnosed with disease recurrence (rT0N3M0, stage IIIC) (Fig. 1A). Following the detection of EGFR L858R mutation (allelic fraction/AF = 2.61%) in tumor biopsy and plasma samples using the 168-gene panel (Burning Rock Biotech, China) next-generation sequencing (Fig. 1B), the patient initiated first-line treatment with gefitinib (250 mg daily (qd) by mouth (po)). The best objective response was stable disease according to Response Evaluation Criteria in Solid Tumors (RECIST) 1.1 criteria. In Sep 2020, the patient developed progressive disease with new liver lesions identified by abdominal CT scan (Fig. 1A). Upon gefitinib progression, next-generation sequencing was conducted on the patient's plasma sample, revealing the persistence of EGFR L858R mutation (AF = 0.14%) (Fig. 1B). Subsequently, the patient transitioned to a second-line regimen of chemotherapy (pemetrexed and carboplatin) combined with bevacizumab. However, a CT scan showed pulmonary oligoprogressive lesions after a progression-free survival of four months (Fig. 1A). After consultation with the radiotherapy department, the patient was treated with stereotactic body radiotherapy. After that, she continued the regimen of chemotherapy combined with bevacizumab. Around three months later, the patient developed multiple new metastatic pulmonary lesions (Fig. 1A), accompanied by a decline in performance status (scored as 2). In June 2021, the patient initiated third-line treatment with chemotherapy (albumin-bound paclitaxel) combined with a programmed death-1 inhibitor. The best objective response was stable disease. After a progression-free survival of over six months, the disease recurred with new brain metastatic lesions (Fig. 1A). Due to the remarkable advantage of furmonertinib's potent blood–brain barrier penetration ability,^3^ the patient was subsequently treated with furmonertinib (160 qd po) with a progression-free survival of 14 months. Partial response was confirmed at one month for both pulmonary and brain lesions. Repeated liquid biopsies of plasma revealed EGFR L858R (AF = 8.92%) and EGFR C797S (AF = 11.43%) (Fig. 1B) when the disease progressed (Fig. 1A). Given this outcome, we decided to treat our patient with furmonertinib (160 qd po) and erlotinib (150 qd po). The best objective response was stable disease. However, the patient achieved a progression-free survival of only five months, and the best objective response was stable disease. Next-generation sequencing was performed with the plasma sample at progressive disease (Fig. 1A) and revealed EGFR L858R mutation (AF = 2.61%), C797S mutation both in cis (AF = 1.82%) and in trans (AF = 0.34%) with T790M mutation (AF = 1.86%), and EGFR L718Q mutation (AF = 0.92%) (Fig. 1B). Case reports and retrospective analyses could demonstrate the significant efficacy of afatinib in the treatment of patients with EGFR L858R and L718Q mutations after third-generation EGFR-TKI resistance.^4^^,^^5^ Based on the previous studies, a three-drug synergistic targeted drug combination, consisting of erlotinib (150 qd po), furmonertinib (160 qd po), and afatinib (30 qd po), was administered to the patient. The best objective response was stable disease.

**Figure 1:**
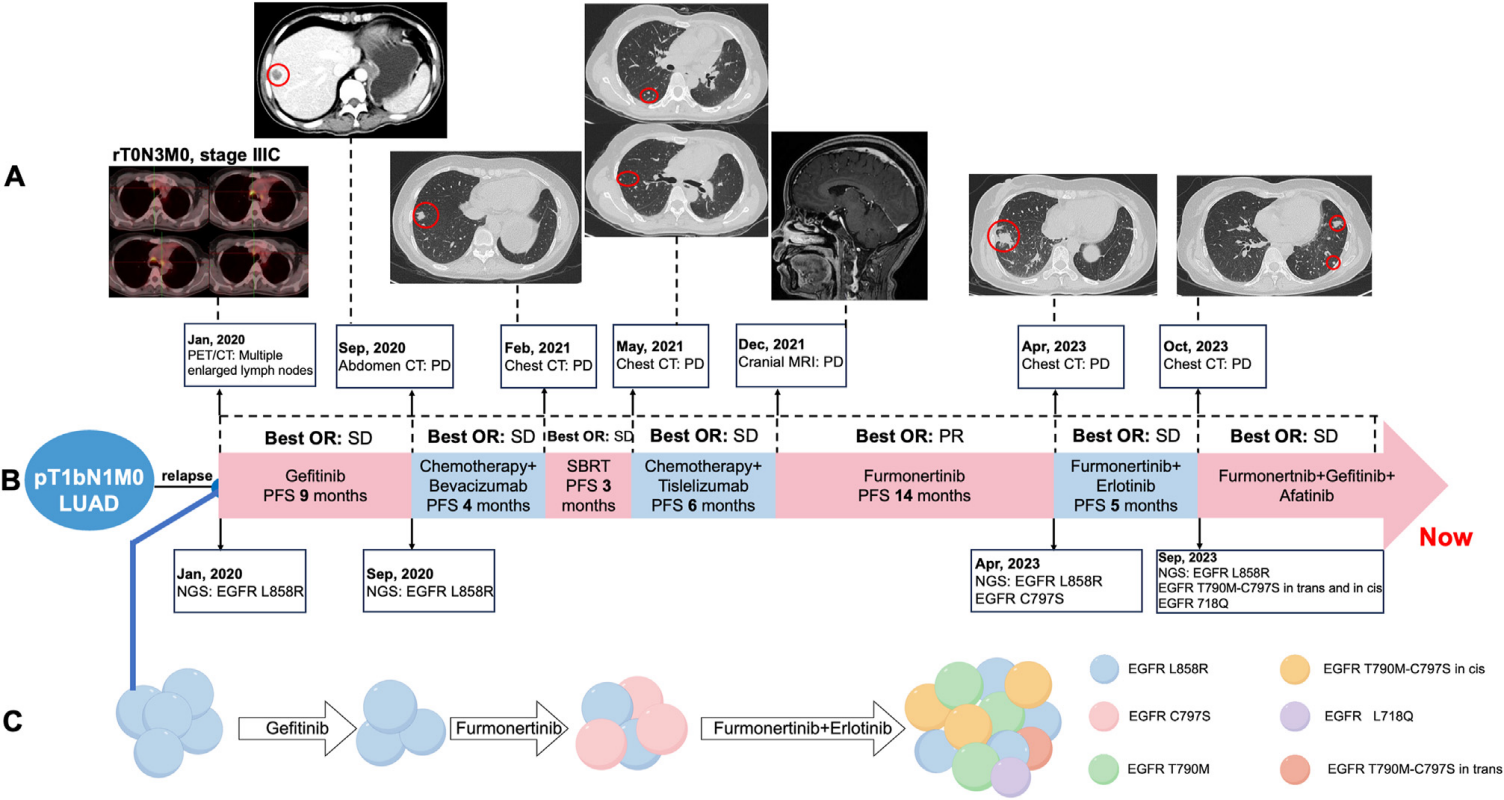
The patient's diagnostic and treatment history. **(A)** Image maps of each progressive disease (PD) time point. **(B)** The therapeutic regimens received, the associated best objective response (OR), progression-free survival (PFS), and the major mutations detected. **(C)** Conceptual models of genomic evolution. LUAD, lung adenocarcinoma; MRI, magnetic resonance imaging; NGS, next-generation sequencing; PET-CT, positron emission tomography–computed tomography; PR, partial response; SD, stable disease.

To our knowledge, this is the first case report that provides clinical evidence of the combination use of erlotinib, furmonertinib, and afatinib for resolving EGFR T790M-C797S both in cis and in trans and EGFR L718Q resistant mutation. For our case, we believe that the modest efficacy of a three-drug synergistic targeted drug combination is attributable to clonal heterogeneity (Fig. 1C shows the patient's clonal progression). Due to clonal heterogeneity, patients with non-small cell lung cancer carry changing EGFR mutations, so preventive interventions before the emergence of EGFR-TKI resistance are clinically crucial, such as therapeutic strategies that combine EGFR-TKIs with chemotherapy or antivascular agents, and dual-targeted therapies, which have been found to prolong treatment responses in patients with non-small cell lung cancer.

## Ethics declaration

This study was conducted in accordance with the Declaration of Helsinki and was approved by the Ethics Committee of Zhejiang Cancer Hospital (IRB-2023-378). No human experimentation is involved in this letter.

## Funding

This study was supported by the Natural Scientific Foundation of Zhejiang Province, China (No. LTGY23H160007).

## CRediT authorship contribution statement

**Kaibo Ding:** Conceptualization, Writing – original draft. **Zhongsheng Peng:** Data curation. **Yanjun Xu:** Conceptualization, Writing – review & editing.

## Conflict of interests

The authors declare that they have no known competing financial interests or personal relationships that could have appeared to influence the work reported in this paper.
