# Supplementary material for: Triplet therapy overcomes 3rd-EGFR TKI-resistant EGFR-L858R/T790M/C797S in trans and in cis/L718Q mutation
Source: Genes Dis. 2024 Sep 7;12(2):101408. doi: 10.1016/j.gendis.2024.101408 (PMC11742358; doi:10.1016/j.gendis.2024.101408)
Supplement: Multimedia component 1 [file mmc1.docx]

**Material and Methods**

**Platform and Content:**

We employed a 168-gene panel provided by Burning Rock Biotech, China, for next-generation sequencing (NGS). This comprehensive panel covers 168 genes known to be associated with various cancers, including EGFR, allowing for the simultaneous identification of multiple mutations. The panel is designed to detect a wide range of genetic alterations, such as single nucleotide variants (SNVs), insertions/deletions (InDels), copy number variations (CNVs), and gene fusions.

**Sample Preparation and Sequencing:**

As part of the routine clinical workflow, formalin-fixed, paraffin-embedded (FFPE) tumor tissue and/or peripheral blood samples were submitted for NGS testing at the request of the attending physician. NGS testing was conducted in a clinical diagnostic laboratory certified by the Clinical Laboratory Improvement Amendments (CLIA) and the College of American Pathologists (CAP).

For the FFPE tumor tissue, 4 μm sections of 10–15 unstained slides and one H&E-stained section were cut from the tissue block. The H&E-stained section was used to evaluate the cellularity of the FFPE tissue, and sections containing at least 200 tumor cells and a tumor cell fraction ≥20% were deemed suitable. Tumor DNA (tDNA) was extracted from the unstained slides using the QIAamp DNA FFPE Tissue Kit (Qiagen, Düsseldorf, Germany) according to the manufacturer’s instructions.

Peripheral blood samples (8 ml) were collected in ethylenediaminetetraacetic acid (EDTA) tubes. Circulating cell-free DNA (cfDNA) was extracted from 4 to 5 ml of blood plasma using the QIAamp Circulating Nucleic Acid Kit (Qiagen, Düsseldorf, Germany) within two hours of sample collection. DNA concentration was measured using a Qubit 2.0 Fluorometer with the Qubit dsDNA HS assay kit (Life Technologies, CA, USA). A minimum of 30 ng of tDNA is required for tissue-NGS library construction, and a minimum of 20 ng of cfDNA is required for plasma-NGS library construction. The presence of ctDNA was confirmed by the detection of any genetic alterations in cfDNA. Tissue DNA or cfDNA was sheared to fragment sizes of 200–400 base pairs using an M220 ultrasonicator (Covaris, MA, USA), followed by end repair, adaptor ligation, and purification. Both tissue-NGS and plasma-NGS were performed using a commercial panel consisting of 168 genes related to lung cancer, covering approximately 160 kb of human genomic regions. The probes in the NGS panel covered critical exons and introns among 168 genes, including 10 frequently rearranged regions.

**Data Analysis and Interpretation:**

Targeted sequencing was conducted on a NextSeq 500 sequencer (Illumina, San Diego, USA) at a coverage depth of 1000× for tissue samples and 10,000× for plasma samples. Sequencing data were mapped to the reference human genome (hg19) using Burrows-Wheeler Aligner (version 0.7.10). Variant identification, annotation, and prioritization were implemented using bioinformatics tools.
